# Not all smokers are alike: the hidden cost of sustained attention during nicotine abstinence

**DOI:** 10.1038/s41386-022-01275-8

**Published:** 2022-01-28

**Authors:** Harshawardhan U. Deshpande, John R. Fedota, Juan Castillo, Betty Jo Salmeron, Thomas J. Ross, Elliot A. Stein

**Affiliations:** 1 Neuroimaging Research Branch, National Institute on Drug Abuse-Intramural Research Program, National Institutes of Health, Baltimore, MD USA; 2 Department of Psychology, Harvard University, Cambridge, MA USA; 3 Present Address: Behavioral and Cognitive Neuroscience Branch, Division of Neuroscience Behavior, National Institute on Drug Abuse, National Institutes of Health, Bethesda, MD USA

**Keywords:** Attention, Addiction

## Abstract

Nicotine Withdrawal Syndrome (NWS)-associated cognitive deficits are notably heterogeneous, suggesting underlying endophenotypic variance. However, parsing this variance in smokers has remained challenging. In this study, we identified smoker subgroups based on response accuracy during a Parametric Flanker Task (PFT) and then characterized distinct neuroimaging endophenotypes using a nicotine state manipulation. Smokers completed the PFT in two fMRI sessions (nicotine sated, abstinent). Based on response accuracy in the stressful, high cognitive demand PFT condition, smokers split into high (HTP, *n* = 21) and low task performer (LTP, *n* = 24) subgroups. Behaviorally, HTPs showed greater response accuracy (88.68% ± 5.19 SD) vs. LTPs (51.04% ± 4.72 SD), independent of nicotine state, and greater vulnerability to abstinence-induced errors of omission (EOm, *p* = 0.01). Neurobiologically, HTPs showed greater BOLD responses in attentional control brain regions, including bilateral insula, dorsal ACC, and frontoparietal Cx for the [correct responses (–) errors of commission] PFT contrast in both states. A whole-brain functional connectivity (FC) analysis with these subgroup-derived regions as seeds identified two circuits: Precentral Cx↔Insula and Insula↔Occipital Cx, with abstinence-induced FC strength increases seen only in HTPs. Finally, abstinence-induced FC and behavior (EOm) differences were positively correlated for HTPs in a Precentral Cx↔Orbitofrontal cortical circuit. In sum, only the HTP subgroup demonstrated sustained attention deficits following 48-hr nicotine abstinence, a stressor in dependent smokers. Unpacking underlying smoker heterogeneity with this ‘dual (task and abstinence) stressor’ approach revealed discrete smoker subgroups with differential attentional deficits to withdrawal that could be novel pharmacological/behavioral targets for therapeutic interventions to improve cessation outcomes.

## Introduction

Following acute smoking abstinence, most smokers exhibit components of the Nicotine Withdrawal Syndrome (NWS), manifest as a set of aversive affective, somatic, and cognitive disruptions peaking in the initial days of a quit attempt [1]. The timing and severity of the NWS symptoms are salient to and predictive of long-term smoking cessation, with more symptoms generally associated with lower success [2, 3]. Indeed, the NWS often dissuades smokers from continued abstinence through negative reinforcement, thus promoting relapse to alleviate the withdrawal state [4–6].

The clinical symptoms of the NWS vary greatly in their duration, intensity, and phenomenology across individuals [7–9]. Indeed, only some smokers even report abstinence-induced overt cravings, attentional lapses, and anxiety [10]. Moreover, the efficacy of current FDA approved cessation aids such as bupropion [11], varenicline and nicotine replacement therapy (NRT [12]) is quite variable, suggesting heterogeneity in the smoking phenotype. Further, genetically informed biomarkers such as the nicotine metabolite ratio induced by variations in the hepatic enzyme cytochrome P450 (CYP2A6 [13]) predict success with NRT and lend further credence to the underlying population diversity. Taken together, these studies indicate considerable variance related to NWS symptom duration, intensity, and cessation outcomes in the aggregate smoker population.

One way to partition this variance and reveal heterogeneity amongst smokers is the induction of multiple stress mechanisms. Chronic stress from such mechanisms likely results in a maladaptive response known as allostatic load [14–16]. Such longstanding elevated activation of the brain’s stress systems during nicotine abstinence likely reduces smokers’ ability to appropriately respond when presented with additional extrinsic stressors, thus enhancing the reinforcing effects of returning to nicotine use, and increasing relapse vulnerability [5, 17]. For example, while smoking cue reactivity or nicotine abstinence effects may not predict relapse on their own, a recent study showed successful prediction of relapse when they were combined [18].

Cognitively demanding tasks are also known to produce autonomic stress-like responses [19, 20]. During nicotine withdrawal, smokers often report impairments in cognitive control, including decrements in performance on tasks requiring sustained attention [21–23], response inhibition [24], and working memory [25–27]. These cognitive deficits are predictive of smoking relapse [28, 29], especially at high levels of task difficulty [25, 30]. Lesage et al. [31] reported attentional deficits (including greater errors of omission, EOm) in acutely abstinent smokers, which were alleviated by transdermal nicotine administration, highlighting dampened sustained attention as a potentially important component of the NWS.

Acute nicotine abstinence is also a stressful experience for smokers [32, 33]. For example, Sutherland and colleagues [34] found that in abstinent (vs. sated) smokers, the resting state functional connectivity (rsFC) strength in an amygdala-insula-Default-Mode Network (DMN) circuit was downregulated following NRT administration, suggesting relief of the reported subjective withdrawal state. Fedota et al. [35] found that dorsal and posterior insular rsFC circuits with the DMN and the Salience Network (SN) are enhanced during abstinence (vs. satiety), while a ventral insular rsFC connection to the Executive Control Network (ECN) was reduced, and time-varying FC changes show reduced temporal flexibility and lower network spatiotemporal diversity between abstinence and satiety [36]. Together, these observations demonstrate the utility of cognitive demand and abstinence manipulations as stressors that reveal changes in rsFC in smokers.

We thus posited that simultaneous allostatic challenges of cognitive demand and nicotine abstinence could partition the variance in stress response of chronic nicotine smokers. In the current study therefore, we leveraged a ‘two-level stress test’ of a cognitively demanding parametric flanker task (PFT) superimposed upon acute abstinence as allostatic stressors to enhance the variability in maladaptive processes and neurobiological mechanisms to: a) fractionate smokers into distinct endophenotypic subgroups and b) characterize the differential behavioral and neurobiological responses of these subgroups to nicotine abstinence. Using a within-subjects design, we hypothesized that subgroups would be identified based on differential cognitive performance and that these subgroups would show differences in task-evoked behavioral attentional lapses, brain activation patterns, and underlying task-related FC patterns precipitated by nicotine withdrawal (vs. satiety).

## Materials and methods

### Participants

Fifty-nine right-handed smokers were recruited into the study. Written informed consent was obtained in accordance with the National Institute on Drug Abuse, Intramural Research Program Institutional Review Board. Fourteen were excluded during final analyses (two for incomplete behavioral data, two for scanning errors, two for abnormal MRI, three due to medical exclusions, and five for excessive head motion). Complete demographic description, nicotine use, and alcohol use for the remaining forty-five participants are described in Table 1. Comorbid psychiatric, neurological, or other drug abuse/dependence was exclusionary. Participants had to present with a negative urine drug or breath alcohol screen on all scan days. Additional exclusion criteria are detailed in the Supplemental methods. Previously published studies included a subset of these participants [35, 37].

**Table 1:** Demographics for the whole cohort (*n* = 45) of smokers and both SUBGROUPs (*n* = 21 for HTP and *n* = 24 for LTP based on adjusted accuracy on the high DEMAND condition of the Parametric Flanker Task).

Variable	Total cohort (*n* = 45)	Task performance subgroups		Test statistic
		High task performers (HTP)	Low task performers (LTP)	
Gender (M/F)	27/18	13/8	14/10	χ^2^ = 0.06, *p* = 0.81
Age (years)	38.9 ± 1.5	39.3 ± 2.0	38.6 ± 2.2	*t* = −1.65, *p* = 0.11
Race (A/AA/C/M)	1/19/21/4	1/12/8/0	0/7/13/4	χ^2^ = 7.34, *p* = 0.06
Education (years)	13.44 ± 0.4	13.2 ± 0.7	13.6 ± 0.5	*t* = −0.13, *p* = 0.90
IQ	105 ± 2.0	101.4 ± 2.9	108.2 ± 2.8	*t* = −2.20, *p* = 0.10
FTND^a^	4.4 ± 0.3	5 ± 0.4	3.4 ± 0.4	*t* = 2.30, *p* = 0.03
Avg cigarettes per day	14.0 ± 0.8	15.0 ± 1.3	13.1 ± 1.0	*t* = −0.99, *p* = 0.33
Age began smoking	17.2 ± 0.8	18.4 ± 1.4	16.1 ± 0.8	*t* = 1.06, *p* = 0.30
Years smoked	18.5 ± 1.6	18.8 ± 2.4	18.3 ± 2.1	*t* = −0.5, *p* = 0.57
Alcohol use (drinks per week, only users)	6.1 ± 0.2	3.33 ± 0.3	7.9 ± 0.4	*t* = 2.03, *p* = 0.08

*A* Asian, *AA* African American, *C* Caucasian, *M* Mixed, *IQ* Intelligence Quotient, *FTND* Fagerström Test of Nicotine Dependence.

^a^denotes a significant group difference. Values represent mean ± standard error.

### Experimental design

**Fig. 1:**
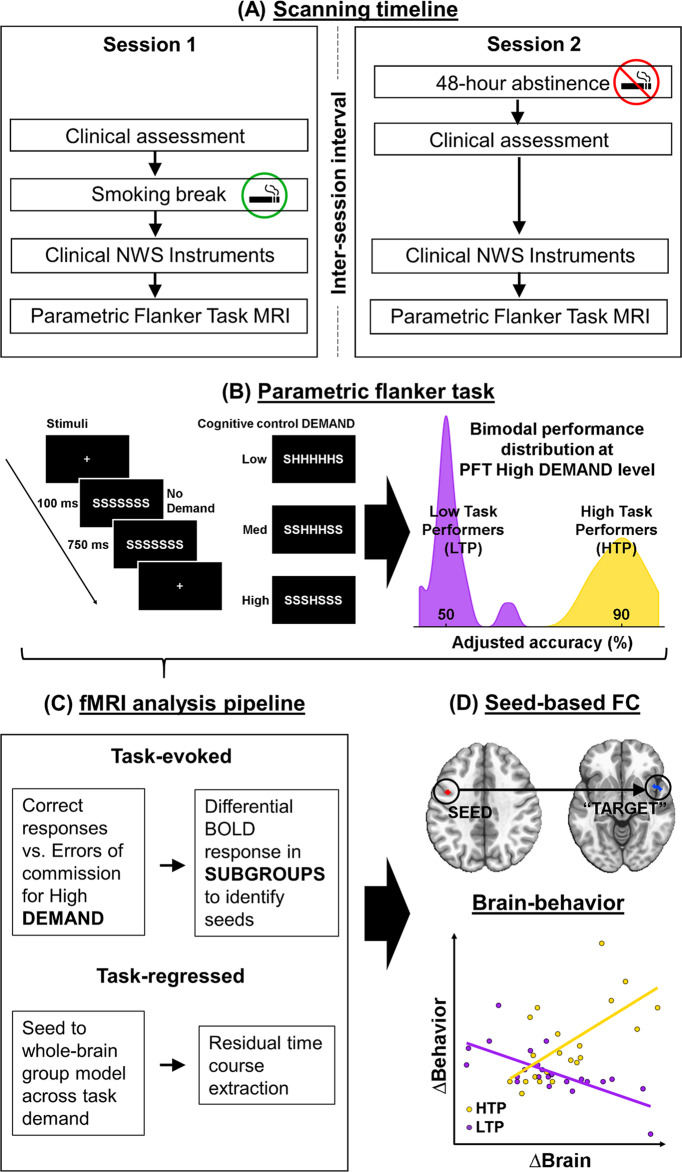
Study experimental design, data analysis pipelines and key findings. **A** In a within-subjects design with two scanning sessions, participants were nicotine sated (session 1) or ~48 hours abstinent (session 2). The inter-session interval averaged at 75 days (median 28 days). **B** During both MRI scans, participants performed a 25-minute Parametric Flanker Task (PFT). Cognitive control DEMAND is modulated via the number of conflicting stimuli flanking the target stimuli (no, low, medium, or high DEMAND). Based on the adjusted accuracy at the high DEMAND, participants were divided into two SUBGROUPs: Low task performers (LTP) and High task performers (HTP). **C** The task-evoked pipeline was used to identify brain regions showing greater differential response in high DEMAND for the [correct responses (–) errors of commission] for HTPs vs. LTPs. Using the task-regressed functional connectivity (FC) pipeline, these regions were used in a seed-based FC analysis (**D**) to identify seed-“target” *dyads* with FC SUBGROUP differences between STATEs. Finally, SUBGROUP STATE differences in FC and Errors of Omission (EOm) were related.

In a longitudinal within-subjects design, participants completed two MR scanning sessions––one during sated smoking and another after ~48 h of biochemically verified nicotine abstinence. The order of the two scan sessions was fixed since these data are part of a larger ongoing smoking cessation protocol (clinicaltrials.gov Identifier: NCT01867411). The sated scan preceding the abstinence scan by an average of 75 days (median 28 days). See Fig. 1 for experimental design overview.

### Behavioral and subjective measures

Participants were scanned while completing a PFT. The PFT was modified from the classic Eriksen flanker task [37, 38] to instantiate varying levels of DEMAND for cognitive control on a trial-by-trial basis (Fig. 1B). All stimuli were presented using E-Prime software (Psychology Software Tools, Sharpsburg, PA).

Subjective ratings of withdrawal (Wisconsin Smoking Withdrawal Scale, WSWS [39]), craving (Tobacco Craving Questionnaire, TCQ-SF [40]) and affect (Positive and Negative Affect Schedule, PANAS [41]) were assessed prior to each scanning session.

The Supplemental methods contain a detailed explanation of the PFT, subjective measurements and MRI acquisition parameters.

### Data analysis

#### Behavioral Task and subjective measures

Behavioral effects of smoking: During PFT performance, STATE (satiety/abstinence), and demand for cognitive control: DEMAND (none/low/medium/high) were quantified via correct response speed (*Speed* = 1/Reaction Time), coefficient of variation of correct response speeds (*SpdCV* = std(Speed_CorrectTrials_)/mean(Speed_CorrectTrials_)), trial adjusted accuracy (*Accuracy* = Correct Trials/(Correct Trials + Errors of Commission(ECo))), and Errors of Omission (EOm)––a measure of lapses in attention. Performance accuracy in the high DEMAND condition identified SUBGROUPs of participants (High Task Performers/Low Task Performers, HTP/LTP). All subsequent behavioral and neuroimaging analyses accounted for these SUBGROUPs. All analyses were conducted using R.

*Speed, SpdCV*, and *Accuracy* were submitted to mixed-design analysis of variance (ANOVA) with STATE and DEMAND as within-subjects factors, and SUBGROUP as a between-subjects factor. Significant interactions were evaluated via post-hoc paired and unpaired t-tests where appropriate. ΔEOm were calculated as a function of STATE (abstinence [−] sated) prior to examination of between-subject SUBGROUP differences via Kruskal-Wallis one-way ANOVA. Significant non-parametric effects were evaluated via post-hoc Wilcoxon rank-sum test.

Assumptions of normality in the subjective measures (WSWS, TCQ, PANAS) were evaluated following calculation of the difference as a function of STATE (Δ Score; abstinence [−] sated). STATE effects were calculated via one-sample *t*-tests comparing Δ Score against null hypotheses of 0; between-subject differences in SUBGROUP identity were evaluated via independent *t*-tests. Where appropriate, equivalent nonparametric statistical tests were utilized.

#### Imaging

Data were collected on two Siemens scanners (Trio, *n* = 35 and Prisma, *n* = 10). The PFT fMRI data were processed and analyzed using AFNI [42] in two parallel pipelines: task-evoked activation and task-regressed functional connectivity. The task-evoked pipeline modeled differences between SUBGROUPS (HTP/LTP) in task-evoked responses during the PFT high DEMAND condition. In addition, since attentional lapses (EOm) were observed across all DEMAND conditions, the PFT-evoked neural responses were regressed out in a task-regressed pipeline. The details of these pipelines are described below.

### Task-evoked pipeline

#### Individual level analysis

After preprocessing (see Supplemental methods), an event-related analysis of the PFT data was performed using a voxel-wise multiple regression analysis with regressors expressed as a delta function convolved with a standard hemodynamic response function (gamma variate basis function) and its temporal derivative (*AFNI: 3dDeconvolve*). Regressors included DEMAND (none, low, medium, high) for both correct and error responses (eight total regressors) and six head motion parameters. A voxel-wise average amplitude change equal to the percentage change from baseline (β) was calculated per participant and regressor. The design matrix obtained was applied to the concatenated normalized time series (*AFNI: 3dREMLfit*) to obtain the beta +statistics dataset with restricted maximum likelihood estimation and the dataset for the restricted maximum likelihood residuals. The minimum voxel cluster size for all whole-brain analyses was determined (*AFNI: 3dFWHMx, 3dClustSim*) using a two-component measure of the spatial autocorrelation of the preprocessed data [43].

#### Group level analysis

To identify task activation differences to serve as FC seeds, a multivariate model approach (*AFNI: 3dMVM*) was used with a SUBGROUP contrast (HTP vs. LTP) of correct trials vs. error trials at the high DEMAND condition. Factors in the model were STATE (satiety, abstinence) and SUBGROUP (HTP, LTP) with scanner (TRIO, PRISMA) and ΔFD head motion (abstinent [−] sated) as covariates. A conservative voxelwise threshold of *p* = 0.0001 was applied on the SUBGROUP difference for the high DEMAND correct trials vs. error trials contrast, which identified 19 spatially specific clusters (minimum *k* = 23 voxels; family-wise error of ɑ ≤ 0.05). These clusters were then used in a seed-based FC analysis on the task-regressed BOLD data to identify SUBGROUP FC differences to relate with EOm behavioral data.

### Task-regressed Pipeline

The significant clusters of PFT-evoked activation showing SUBGROUP differences obtained from the above task-evoked pipeline were used as regions-of-interest (ROIs) in a seed-based FC analysis to identify FC SUBGROUP mechanisms. Since attentional lapses (objectively measured as EOms) were not limited to a specific DEMAND condition, their relationship with FC SUBGROUP differences was investigated across all DEMAND conditions. As using task-evoked data to perform seed-based FC analyses can produce false positives and inflate the FC estimates [44], seed-based FC analyses were conducted after regressing out the PFT and modeling the hemodynamic response function (HRF) using a finite impulse response model [45, 46]. The HRF was modeled as a set of tent functions (eight parameter TENT function for 14 time points, *AFNI: 3dDeconvolve, 3dREMLfit*). The residual time series was used as a proxy for resting-state data to characterize differences between SUBGROUP FC and the relationship of SUBGROUP FC to EOm. Data were band pass filtered between 0.001 Hz and 0.25 Hz (*AFNI: 3dTproject*).

### Seed-based FC

While the SUBGROUPs were revealed by behavioral *Accuracy* on the high DEMAND PFT condition, a SUBGROUP*STATE effect on *EOm* was observed across all task conditions. To characterize the network mechanisms underlying this decrease in sustained attention in the HTP SUBGROUP, a multivariate model (*AFNI: 3dMVM*) was created for each of the observed 19 ROIs which served as FC seeds; whole-brain FC was examined for a SUBGROUP main effect and a SUBGROUP*STATE interaction. A voxelwise threshold of *p* = 0.001 (*k* = 69 voxels; FWE of *ɑ* ≤ 0.05) was used to identify clusters with a SUBGROUP*STATE interaction from each seed to the entire brain. Pairs of regions with significant FC interaction after voxelwise and cluster thresholding are denoted as *dyads*, with the task activation difference pole denoted as the “seed” and the differential FC region denoted as the “target” pole. The “target” pole was subsequently used as a seed in a second whole-brain FC analysis to identify regions with which it had differential functional connections (e.g., Sutherland et al. 2013a [34]) to define larger functional circuits differing between HTPs and LTPs.

### Relating STATE differences in functional connectivity (ΔFC) with behavioral attentional lapses (ΔEOm)

To relate FC STATE differences with changes in behavior relevant to EOm, a multiple linear regression analysis was modeled (*AFNI: 3dRegAna*). The model included ΔEOm, SUBGROUP membership and a SUBGROUP*ΔEOm interaction term. ΔFD was included as a covariate to account for residual motion. Since six separate regression analyses were conducted, a threshold of *p* = 0.001 (*k* = 100 voxels; FWE of ɑ ≤ (0.05/6)) was used to account for multiple comparisons.

## Results

### Cohort behavioral and subjective measures

There was a main effect of DEMAND, with lower Speed, SpdCV and Accuracy for the high DEMAND condition and a main effect of STATE, with Accuracy lower and EOm higher during nicotine abstinence (vs. satiety). As expected, subjective ratings of withdrawal, craving and affect also showed a main effect of STATE. See Supplemental results, Table ST1, and Table ST2 for detailed behavioral/subjective cohort results.

### Subgroup behavioral and subjective differences

**Fig. 2:**
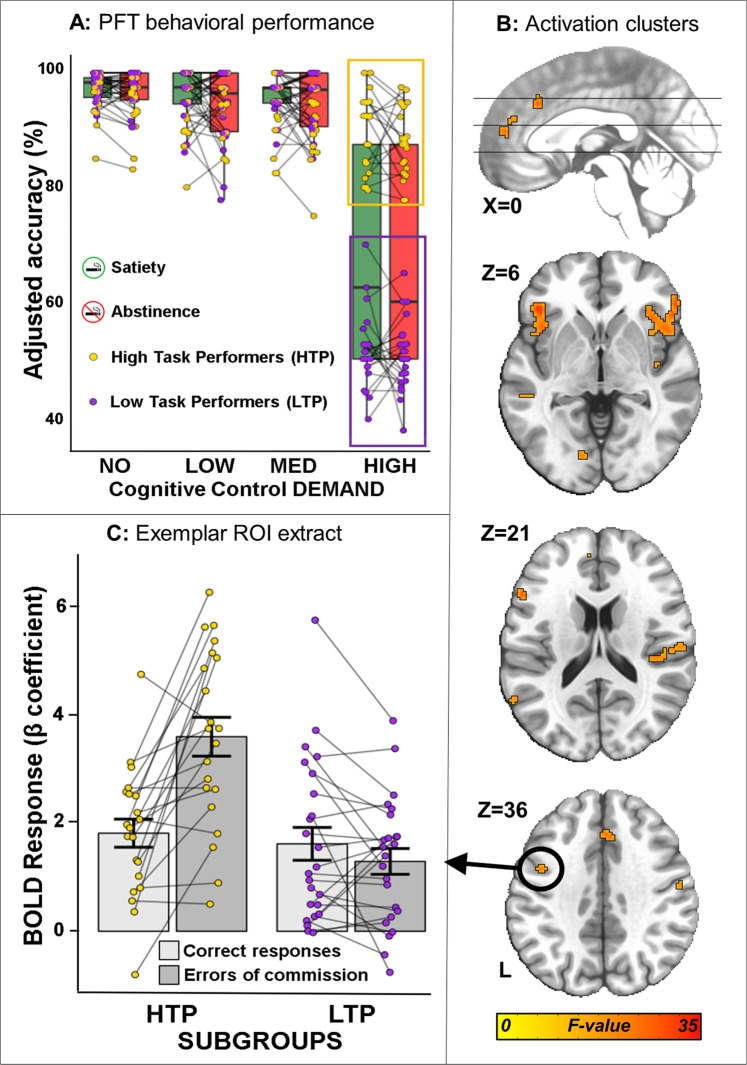
Behavioral and neurobiological (BOLD response) differences between SUBGROUPs on PFT responded trials across nicotine STATE. **A** In both satiety (green) and abstinence (red), High task performers (HTP, yellow box, 88.68% ± 5.19 SD) show higher accuracy only in the High DEMAND condition compared to Low task performers (LTP, purple box, 51.04% ± 4.72 SD) for responded trials. **B** PFT-evoked BOLD response clusters with differential activation for the HTP compared to the LTP SUBGROUP for the [correct responses (–) errors of commission] trials contrast at the high DEMAND condition across STATE. These clusters were subsequently used as seeds in a whole-brain seed-based functional connectivity analysis. **C** Extracted β coefficients from the encircled cluster (averaged across STATE) showing greater sensitivity to errors of commission vs. correct responses in the HTP SUBGROUP and no difference between correct responses and errors of commission for the LTP SUBGROUP. This region has been chosen as an exemplar. For ROI extracts from all clusters see Fig. S3. HTP High Task Performers, LTP Low Task Performers, ROI Region of Interest.

A clear dichotomy was observed in Accuracy in the high DEMAND PFT condition across nicotine STATEs, which we used to define two SUBGROUPs: High task performers (HTP, *N* = 21) with 88.68% (±5.19 SD) Accuracy and Low task performers (LTP, *N* = 24) with 51.04% (±4.72 SD) Accuracy (Fig. 2A). Speed decreased with task DEMAND for both SUBGROUPs (Supplemental Table ST2, Fig. SF2). A virtually identical performance profile and behavior-derived subgrouping was also observed in a separate group of age-matched healthy controls (*N* = 31) with no previous nicotine exposure, demonstrating that this task performance subgroup separation is not specific to the smoker cohort in the study or previous/current drug usage (see Supplemental Fig. SF3).

#### Demographics

Perhaps counterintuitively, the HTPs had greater nicotine dependence (*p* = 0.03; FTND score for HTPs 5 ± 0.38 SD) compared to the LTPs (3.83 ± 0.35 SD)). No other demographic measures, nicotine use, or alcohol use differed between the SUBGROUPs (Table 1).

#### Task performance

**Fig. 3:**
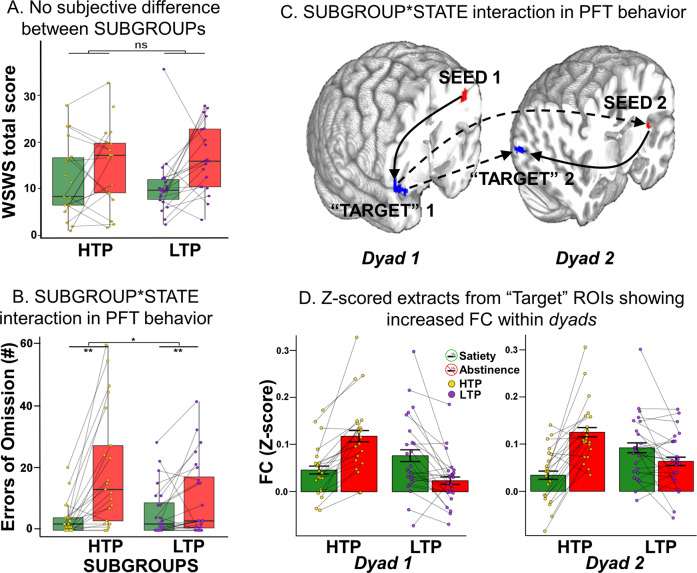
SUBGROUP*STATE differences in behavior and functional connectivity (PFT-regressed fMRI). **A** There was no difference between the HTP and LTP SUBGROUPs for the total score of the subjective WSWS (*p* = 0.29). **B** Across all demand conditions, HTPs (vs. LTPs) show a greater increase (Kruskall–Wallis chi-squared = 5.4741, *p* = 0.01) in the number of Errors of Omission (EOm) during abstinence; HTP: *W* = 378, *p* = 8.76e-06, LTP: *W* = 432, *p* = 9.47e-4. **C** *Dyad1* with the L Precentral seed (L Pre, red) and the R vent Insula “target” (RvI, blue) and *dyad2* with the L pos Insula seed (LpI, red) and the R mid Occipital “target” (RmO, blue). A “target” in our analysis was defined as a significant cluster arising from a whole-brain seed-based FC analysis. The solid arrows indicate within-*dyad* FC while the dotted arrows indicate between-*dyad* FC. The arrows for seed- “target” and *dyad*-*dyad* FC show do not imply directionality or causality. **D** Z-scored FC averages showing increased FC during nicotine abstinence (red bars, SUBGROUP*STATE interaction) within *dyad1* (left, LPre↔RvI) and *dyad2* (right, LpI↔RmO). Error bars show standard error of the mean. Please see Supplemental Table ST4 for “seed” and “target” MNI coordinates. HTP High Task Performers, LTP Low Task Performers, WSWS Wisconsin Smoking Withdrawal Scale, L Pre Left Precentral gyrus, RvI Right ventral Insula, LpI Left posterior Insula, RmO Right middle Occipital, MNI Montreal Neurological Institute.

There was a significant SUBGROUP*STATE ΔEOm effect, with more attentional lapses in the HTPs (Fig. 3B, Kruskall–Wallis chi-squared = 5.4741, df = 1, *p* = 0.01). Both SUBGROUPs had an increase in EOm during abstinence (HTP: *W* = 378, *p* = 8.76e-06, LTP: *W* = 432, *p* = 9.47e-4). No other SUBGROUP, SUBGROUP*STATE or SUBGROUP*DEMAND effects were observed for the Speed, SpdCV, and EOm task performance metrics (Supplemental Table ST2, Fig. SF2).

#### Subjective measurements

No SUBGROUP differences in subjective measures were found, including withdrawal, craving, and affect nor any SUBGROUP*STATE interactions (Fig. 3A). Both groups showed the expected STATE effects (higher negative and lower positive values in clinical instruments). See Supplemental results, Table ST1, and Fig. SF1 for detailed subjective results.

#### Neuroimaging

Based on our hypotheses, our analyses focused on neuroimaging SUBGROUP differences. See Supplemental Fig. SF4 for the overall PFT task map.

### Subgroup differences in the high DEMAND condition (Correct – ECo)

Based on SUBGROUP difference on task accuracy behavior, we identified task-evoked regional brain differences using the high DEMAND contrast trials [correct responses (−) errors of commission] (Fig. 2B, p-voxel < 0.001; p-corrected < 0.05) between SUBGROUPs and across nicotine STATE. Only the HTP SUBGROUP showed a larger BOLD response for error vs. correct trials in regions including bilateral insula, dorsal ACC, frontoparietal attentional regions and right thalamus (exemplar ROI extract in Fig. 2C). Activated regions and BOLD signal ROI values are listed in the Supplement (Table ST3, Fig. SF5).

### Subgroup differences in seed-based FC (task-regressed analyses)

To identify network communication during task performance, the 19 clusters derived from the PFT-evoked differences described above were next used as seeds in 19 independent whole-brain FC analyses. These analyses interrogated the relationship between FC and the behavioral SUBGROUP*STATE effects observed in EOm (objective measurement of attentional lapses) across all PFT DEMAND conditions. Two of the 19 ROIs employed as seeds showed a SUBGROUP*STATE interaction (Fig. 3C, p-voxel < 0.001; p-corrected < 0.05). Specifically, the L Precentral (LPre) seed with R ventral insula (RvI), while the L posterior insula (LpI) seed showed a SUBGROUP*STATE interaction (Fig. 3C, p-voxel < 0.001; p-corrected < 0.05) with the R Mid Occipital (RmO) region. Both circuits showed increased FC (extracted averaged Z-scored FC in Fig. 3D) in the HTPs during abstinence but no significant change for the LTPs, established by separate analyses for each SUBGROUP. These identified circuits (i.e., LPre↔RvI and LpI↔RmO) are denoted as *dyads* 1 and 2, respectively, with one pole of the *dyad* being the seed and the other pole being the functionally connected ‘target’.

Finally, we employed an additional method of separating the participants based on task adjusted accuracy (see Supplemental methods), i.e., rather than overall (averaged) performance level, the correlation between the difference in performance between states (∆Accuracy, sated (−) abstinent) with the difference in the functional connectivity brain measurement between states (∆FC, sated (−) abstinent) was examined. No significant correlations were found for either of the *dyad* circuits (*p* = 0.49 for *dyad1*, *p* = 0.81 for *dyad2*, see supplemental results and supplemental Fig. SF7) supporting the subgroup separation based on overall (averaged) performance level.

### FC coactivation between *dyad1* and *dyad2*

Each of the ‘target’ poles from the initial task FC analyses (*dyads* 1 and 2) was then used as a seed in a second SUBGROUP*STATE whole-brain FC analysis to further identify network interaction differences between the SUBGROUPs.

*Dyad1* (LPre↔RvI): Increased FC is observed between the RvI and LpI for the HTPs during abstinence, indicating functional coactivation between the RvI (*dyad1* pole) and the LpI (*dyad2* pole).

*Dyad2* (LpI↔RmO): We observed increased FC between the RmO and RvI for the HTPs during abstinence, indicating functional coactivation between the RmO (*dyad2* pole) and the RvI (*dyad1* pole).

Thus, the pair of *dyads* appeared to be functionally coactivated under the above conditions (Fig. 3C, dashed lines).

### Brain-behavior interactions: SUBGROUP FC brain differences with attentional lapses (EOm)

**Fig. 4:**
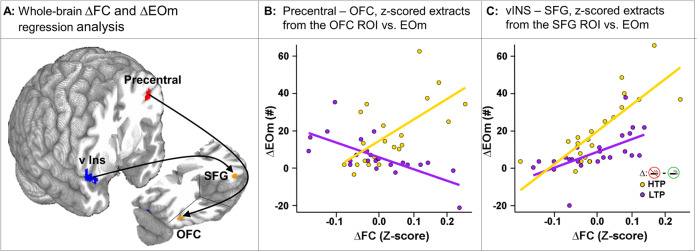
Brain (∆FC) and behavior (∆EOm) regression between SUBGROUP*STATE differences (abstinence – satiety) in individual Z-scored FC extracts (averaged over “target” ROI) and in EOm across all DEMAND levels of the PFT. **A** Whole-brain regression analyses starting with seeds at the L Precentral (L Pre, red) and R ventral Insula (RvI, blue) and “targets” in the R orbitofrontal cortex (R OFC) and L superior frontal gyrus (L SFG) respectively (orange). The arrows do not imply directionality or causality. **B** The z-score FC extract difference (abstinence – satiety) averaged over the R orbitofrontal cortex vs. the difference in the number of EOm for all DEMAND levels of the PFT. The HTPs show a *positive* relationship between the ∆FC and ∆EOm while the LTPs show a *negative* relationship for the L Pre↔R OFC. **C** The z-score FC extract difference (abstinence – satiety) averaged over the L SFG vs. the difference in the number of EOm for all DEMAND levels of the PFT. Both SUBGROUPs show a *positive* relationship between the ∆FC and ∆EOm for the R v Ins↔L SFG. L Pre Left Precentral gyrus, RvI Right ventral Insula, R OFC Right Orbitofrontal Cortex, L SFG Left Superior Frontal Gyrus, FC Functional connectivity, EOm Errors of Omission.

The relationship between the above SUBGROUP FC difference and the key behavioral SUBGROUP*STATE behavioral difference, i.e., EOm, was characterized using a multiple linear regression model. The seeds of this analysis were derived from the poles of the two *dyads* identified in the above task-regressed analysis. To test the SUBGROUP*STATE relationship, ΔEOm (abstinent [−] sated behavioral metric) and ΔFC (abstinent [−] sated brain metric) we performed correlational analyses for each SUBGROUP (Fig. 4A). Using the LPre in *dyad1* as a seed, there was a significant *positive* relationship between ΔEOm and ΔFC with the R orbitofrontal cortex for the HTPs and a *negative* relationship for the LTPs (Fig. 4B). Additionally, using the RvI in *dyad1* as a seed, there was a *positive* correlation between ΔEOm and ΔFC with the L Medial Frontal cortex for both the HTPs and the LTPs as a main effect of ΔEOm (Fig. 4C). Similar *positive* relationships between ΔEOm and ΔFC for both the HTPs and the LTPs were observed using the RmO in *dyad2* as a seed with regions including R parahippocampal, L Middle Temporal and R Lingual gyri (main effect of ΔEOm, Supplemental Fig. SF6, Table ST5).

## Discussion

The current study consistently identified endophenotypic differences at multiple behavioral and neurobiological levels in an otherwise apparent homogeneous cohort of active smokers using a ‘dual-stressor’ framework. The cognitive stressor (Parametric Flanker Task; PFT) first identified two smoker SUBGROUPs (i.e., High and Low Task Performers, HTP/LTP) based on response accuracy on the high DEMAND task condition, independent of nicotine STATE. Additionally, the imposition of nicotine abstinence as a second stressor revealed a SUBGROUP*STATE behavioral effect of greater sustained attentional lapses (i.e., increased errors of omission, EOm) in the HTPs. This behavioral SUBGROUP*STATE interaction was accompanied by neurobiological alterations evident in both PFT-evoked activation SUBGROUP differences and functional connectivity network-level SUBGROUP*STATE interactions between ROIs associated with attentional control. Taken together, these objectively observed differences at multiple levels of inquiry strongly suggest the presence of distinct SUBGROUPs within the smoker population. Such SUBGROUPs may help explain, at least in part, previous inconsistencies in reported cognitive disruptions following acute abstinence.

Typically, the clinical presentation of the NWS is quantified using various smoker subjective experiences (e.g., withdrawal [39], craving [40] and affect [41]). Some of these measures showed a difference between nicotine satiety and abstinence for the smoker cohort (supplemental results), indicating successful STATE manipulation. However, these measurements showed no SUBGROUP differences (exemplar in Fig. 3A). Contrary to subjective measurements, when sustained attention was objectively measured (EOm), the HTP SUBGROUP showed greater abstinence-induced increases in attentional lapses (Fig. 3B). The HTP SUBGROUP thus suffered from an abstinence-induced sustained attention vulnerability, of which they were not overtly aware, and was only revealed by the ‘dual-stressor’ framework. Without the identification of SUBGOUPs via objective PFT performance, the SUBGOUP*STATE effect on sustained attention could not be observed.

The behavioral performance of the SUBGROUPs identified by the PFT was differentiated by two cognitive components: (1) selective attention (maximizing response accuracy), independent of nicotine STATE and (2) sustained attention (minimizing EOm), which displayed a SUBGROUP*STATE interaction. Electrophysiological indices of cognitive control processes have validated the Flanker paradigm as a modulator of visual selective attention [47, 48]. Maintaining high accuracy on responded PFT trials requires an effortful allocation of resource-limited, fatigue-prone selective attention mechanisms to suppress target-irrelevant information [31]. In a similar vein, sustained attention is also effortful [49] and the PFT has previously been used to elicit EOm via the temporary depletion of neural control resources [50]. Our parametric instantiation of the Flanker task (25 min with a button press required on every trial) thus taxed both attentional control and sustained attention resources.

Based on the above behavioral differences between the SUBGROUPs, the underlying neurobiology described herein helps elucidate the mechanisms through which these SUBGROUPs differ. During task performance in the high DEMAND condition, increased activation of multiple brain regions, including the bilateral insula, dorsal anterior cingulate cortex (dACC), right thalamus and the frontoparietal attentional network was observed in the HTPs compared to the LTPs (Fig. 2B, Table ST3) across STATE. These brain regions are canonically associated with performance monitoring and sustained attention as observed in contrasts of correct vs incorrect trials in the PFT [51]. The dACC and insula also show significantly greater BOLD responses for aware vs. unaware errors in a response inhibition task [52]. Better performance monitoring through the recruitment of the dACC and insula in the HTPs may in part relate to their better response accuracy. Importantly, these task-evoked effects in the HTPs were independent of nicotine STATE—and thus not related to nicotine withdrawal but presumably indicative of enhanced performance monitoring in the HTP SUBGROUP, while LTPs, who performed at chance levels, showed lower recruitment of these areas.

To elucidate circuits differentially related to attentional control in the two SUBGROUPs we next examined FC pattern differences using the above differential activation regions as seeds. Across all 19 seeds tested, only two identified *dyads*: L Precentral↔R ventral Insula (LPre↔RvI) and L posterior Insula↔R middle Occipital (LpI↔RmO) showed a SUBGROUP*STATE interaction such that only the HTPs demonstrated increased FC during nicotine abstinence between the *dyad* nodes. The RvI and LpI are both components of the SN, which plays a key role in monitoring interoception and regulating body homeostasis [34, 53, 54]. Given the key roles played by LPre, RvI, LpI and RmO in visuospatial attentional control [55, 56], the increased FC strength between the *dyad* poles likely contributed to the HTPs ability to selectively attend to high DEMAND PFT trials during abstinence. However, since these regions are also crucial for sustained attention [57, 58], the expenditure of attentional resources on attentional control may have left the HTPs susceptible to sustained attentional lapses when nicotine abstinent. In contrast, the LTPs did not show increases in FC strength between the *dyad* poles and remained relatively impervious to perturbations of sustained attention during nicotine abstinence. Maintenance of attentional control during nicotine abstinence thus may come at the expense of dysregulated sustained attention in the HTPs.

A regression analysis on the four *dyad* poles with behavioral responses for the two SUBGROUPs identified a second set of circuits (Fig. 4A) showing a direct relationship between abstinence-induced brain FC changes (∆FC) and PFT sustained attentional changes (∆EOm). While both SUBGROUPs show a positive relationship between ∆FC and ∆EOm for the RvI↔LSFG connection (Fig. 4C), the correlation of ∆FC with ∆EOm appears stronger in the HTPs (vs. LTPs). In a previous study, the LSFG showed increased BOLD response during attentional lapses compared to correct trials in healthy participants performing a Continuous Performance Task [59]. The direct positive relationship we observed between the ∆FC and ∆EOm across SUBGROUPs in the LSFG suggests its involvement in the suboptimal sustained attention seen in both SUBGROUPs, although to a greater degree in the HTPs. For the LPre↔ROFC circuit, the HTPs showed a positive relationship between ∆FC and ∆EOm (Fig. 4B). Among its attributed functions, the ROFC is associated with spatial selective attention [60] and with target detection [61] during visuospatial attention tasks. In light of our findings, the greater involvement of the ROFC during abstinence suggests better attentional control in the HTPs but coming at the cost of sustained attention. Stronger correlations for the LSFG and ROFC for the HTPs are likely a result of the greater sensitivity to nicotine abstinence in the HTPs, manifest as a greater increase in EOms and increased FC strength in the *dyads* in Fig. 3B. Not only are these brain regions identified in the *dyad* analysis (LPre, RvI, LpI, RmO) and the regression analysis (LSFG, ROFC) associated with attentional control, but they are also related to the NWS via association with smoking cue reactivity [62, 63], nicotine dependence severity [64], and relapse [65].

Although the two stressors (task DEMAND and nicotine STATE) did not interact directly, the attentional demands of the PFT concurrent with the presence/absence of nicotine likely produced a dynamic break with homeostasis in response to allostatic load [16], revealing differential disease-relevant behavioral (attentional lapses) and neurobiological (network FC) SUBGROUP*STATE interactions, i.e., two distinct SUBGROUPs with variable cognitive capacity. A putative smoker endophenotype heterogeneity has been previously implicated through cognitive task response [66], data-driven approaches such as hierarchical clustering on clinical assessment characteristics [67], genetically biased neurobiology [68, 69] and cessation treatment outcomes [12].

Our objective characterization of SUBGROUP*STATE effects in early nicotine abstinence has important clinical and research implications. It is likely that underlying population heterogeneity and compensatory homeostatic mechanisms within smokers could mitigate the detection of robust abstinence-induced cognitive deficits assumed to arise from neuroplasticity-induced changes following chronic nicotine use [70, 71]. Specifically, only small to medium effect size deficits have been reported for attention and WM [72], response inhibition [73], attention and response inhibition [74]. Lesage et al. [75] also reported no effects on inhibition-related BOLD activity, although consistent with the current study, increased EOm in abstinent smokers was observed. Further, the connectivity differences in brain networks specific to sustained attention and NWS may suggest potential differential avenues of treatment interventions, including non-invasive brain stimulation or pharmacological methods, and potentially serve as quantitative biomarkers of successful completion of a course of treatment.

While the within-subjects ‘dual stressor’ design revealed objective differences between smoker SUBGROUPs, there are limitations to consider. The PFT as administered is primarily an attentional control task. While it was also able to index sustained attention, a more direct measurement of sustained attention may reveal further behavioral and neurobiological differences in attentional control between SUBGROUPs. Nevertheless, examining both aspects in one task allowed us to look directly at competition for cognitive resources underlying both constructs. The inter-session interval between scanning sessions was variable (mean 75 days, median 28 days). Since modeling the NWS in the crucial period of acute abstinence was a primary goal of the study, we sacrificed some ecological validity to only scan the smokers when they were 48-hour nicotine abstinent. Additionally, as participants were part of a larger treatment study, counterbalancing of the nicotine STATE manipulation was not possible, i.e., the sated scan always preceded the abstinent one and the cohort of smokers were not recruited with the explicit motive of heterogeneous subgrouping.

In sum, by using concurrent stressors of cognitive demand and nicotine abstinence to allostatically challenge a smoker cohort, we objectively characterized the underlying variance into discrete SUBGROUPs, each with differential susceptibility to abstinence-induced sustained attentional lapses, NWS-related differences in task-evoked brain activation and functional network connectivity. The consistency of these SUBGROUP differences at multiple levels of analysis suggest that this index of smoker heterogeneity may have important clinical utility in predicting smoker susceptibility to NWS-induced cognitive/attentional disruptions, which could lead to targeted treatment interventions by triaging NRT only to a specific subgroup, e.g., those with stronger overall attentional control but weaker sustained attention during abstinence.

## Supplementary information

Supplementary material

## References

[1] Allen SS, Bade T, Hatsukami D, Center B (2008). Craving, withdrawal, and smoking urges on days immediately prior to smoking relapse. Nicotine Tob Res.

[2] Snyder FR, Davis FC, Henningfield JE (1989). The tobacco withdrawal syndrome: performance decrements assessed on a computerized test battery. Drug Alcohol Depend.

[3] Kenford SL, Smith SS, Wetter DW, Jorenby DE, Fiore MC, Baker TB (2002). Predicting relapse back to smoking: contrasting affective and physical models of dependence. J Consult Clin Psychol.

[4] McLaughlin I, Dani JA, De, Biasi M (2015). Nicotine withdrawal. Curr Top Behav Neurosci.

[5] Koob GF, Le Moal M (2008). Review. Neurobiological mechanisms for opponent motivational processes in addiction. Philos Trans R Soc Lond B Biol Sci.

[6] Robinson JD, Li L, Chen M, Lerman C, Tyndale RF, Schnoll RA (2019). Evaluating the temporal relationships between withdrawal symptoms and smoking relapse. Psychol Addict Behav.

[7] Piper ME (2015). Withdrawal: expanding a Key Addiction Construct. Nicotine Tob Res.

[8] Sheets ES, Bujarski S, Leventhal AM, Ray LA (2015). Emotion differentiation and intensity during acute tobacco abstinence: A comparison of heavy and light smokers. Addict Behav.

[9] Perkins KA, Briski J, Fonte C, Scott J, Lerman C (2009). Severity of tobacco abstinence symptoms varies by time of day. Nicotine Tob Res.

[10] Piper ME, Schlam TR, Cook JW, Sheffer MA, Smith SS, Loh W-Y (2011). Tobacco withdrawal components and their relations with cessation success. Psychopharmacology.

[11] Patterson F, Schnoll RA, Wileyto EP, Pinto A, Epstein LH, Shields PG (2008). Toward personalized therapy for smoking cessation: a randomized placebo-controlled trial of bupropion. Clin Pharm Ther.

[12] Lerman C, Schnoll RA, Hawk LW, Cinciripini P, George TP, Wileyto EP (2015). Use of the nicotine metabolite ratio as a genetically informed biomarker of response to nicotine patch or varenicline for smoking cessation: a randomised, double-blind placebo-controlled trial. Lancet Respir Med.

[13] Benowitz N, Swan G, Jacobiii P, Lessovschlaggar C, Tyndale R (2006). CYP2A6 genotype and the metabolism and disposition kinetics of nicotine. Clin Pharmacol Therapeutics.

[14] Fisher S, Reason J. Handbook of Life Stress, Cognition and Health. 1988.

[15] McEwen BS (2000). Allostasis and allostatic load: implications for neuropsychopharmacology. Neuropsychopharmacology.

[16] McEwen BS, Gianaros PJ (2011). Stress- and allostasis-induced brain plasticity. Annu Rev Med.

[17] Richards JM, Stipelman BA, Bornovalova MA, Daughters SB, Sinha R, Lejuez CW (2011). Biological mechanisms underlying the relationship between stress and smoking: state of the science and directions for future work. Biol Psychol.

[18] Allenby C, Falcone M, Wileyto EP, Cao W, Bernardo L, Ashare RL (2020). Neural cue reactivity during acute abstinence predicts short-term smoking relapse. Addict Biol.

[19] Moses ZB, Luecken LJ, Eason JC (2007). Measuring task-related changes in heart rate variability. Conf Proc IEEE Eng Med Biol Soc.

[20] McDuff D, Gontarek S, Picard R (2014). Remote measurement of cognitive stress via heart rate variability. Conf Proc IEEE Eng Med Biol Soc.

[21] Hendricks PS, Ditre JW, Drobes DJ, Brandon TH (2006). The early time course of smoking withdrawal effects. Psychopharmacology.

[22] Hughes JR (2007). Effects of abstinence from tobacco: valid symptoms and time course. Nicotine Tob Res.

[23] Weigard A, Huang-Pollock C, Heathcote A, Hawk L, Schlienz NJ (2018). A cognitive model-based approach to testing mechanistic explanations for neuropsychological decrements during tobacco abstinence. Psychopharmacology.

[24] Harrison ELR, Coppola S, McKee SA (2009). Nicotine deprivation and trait impulsivity affect smokers’ performance on cognitive tasks of inhibition and attention. Exp Clin Psychopharmacol.

[25] Nichols TT, Gates KM, Molenaar PCM, Wilson SJ (2014). Greater BOLD activity but more efficient connectivity is associated with better cognitive performance within a sample of nicotine-deprived smokers. Addict Biol.

[26] Jacobsen LK, Krystal JH, Mencl WE, Westerveld M, Frost SJ, Pugh KR (2005). Effects of smoking and smoking abstinence on cognition in adolescent tobacco smokers. Biol Psychiatry.

[27] Mendrek A, Monterosso J, Simon SL, Jarvik M, Brody A, Olmstead R (2006). Working memory in cigarette smokers: comparison to non-smokers and effects of abstinence. Addict Behav.

[28] Ashare RL, Hawk LW (2012). Effects of smoking abstinence on impulsive behavior among smokers high and low in ADHD-like symptoms. Psychopharmacology.

[29] Patterson F, Jepson C, Loughead J, Perkins K, Strasser AA, Siegel S (2010). Working memory deficits predict short-term smoking resumption following brief abstinence. Drug Alcohol Depend.

[30] Loughead J, Wileyto EP, Ruparel K, Falcone M, Hopson R, Gur R (2015). Working memory-related neural activity predicts future smoking relapse. Neuropsychopharmacology.

[31] Faber LG, Maurits NM, Lorist MM (2012). Mental fatigue affects visual selective attention. PLoS One.

[32] Cleck JN, Blendy JA (2008). Making a bad thing worse: adverse effects of stress on drug addiction. J Clin Invest.

[33] Allenby C, Falcone M, Ashare RL, Cao W, Bernardo L, Wileyto EP (2020). Brain marker links stress and nicotine abstinence. Nicotine Tob Res.

[34] Sutherland MT, Carroll AJ, Salmeron BJ, Ross TJ, Hong LE, Stein EA (2013). Down-regulation of amygdala and insula functional circuits by varenicline and nicotine in abstinent cigarette smokers. Biol Psychiatry.

[35] Fedota JR, Ding X, Matous AL, Salmeron BJ, McKenna MR, Gu H (2018). Nicotine abstinence influences the calculation of salience in discrete insular circuits. Biol Psychiatry Cogn Neurosci Neuroimaging.

[36] Fedota JR, Ross TJ, Castillo J, McKenna MR, Matous AL, Salmeron BJ (2021). Time-varying functional connectivity decreases as a function of acute nicotine abstinence. Biol Psychiatry Cogn Neurosci Neuroimaging.

[37] Fedota JR, Matous AL, Salmeron BJ, Gu H, Ross TJ, Stein EA (2016). Insula demonstrates a non-linear response to varying demand for cognitive control and weaker resting connectivity with the executive control network in smokers. Neuropsychopharmacology.

[38] Forster SE, Carter CS, Cohen JD, Cho RY (2011). Parametric manipulation of the conflict signal and control-state adaptation. J Cogn Neurosci.

[39] Welsch SK, Smith SS, Wetter DW, Jorenby DE, Fiore MC, Baker TB (1999). Development and validation of the Wisconsin Smoking Withdrawal Scale. Exp Clin Psychopharmacol.

[40] Heishman S, Singleton E, Pickworth W (2008). Reliability and validity of a short form of the tobacco craving questionnaire. Nicotine Tob Res.

[41] Watson D, Clark LA, Tellegen A (1988). Development and validation of brief measures of positive and negative affect: the PANAS scales. J Pers Soc Psychol.

[42] Cox RW (1996). AFNI: software for analysis and visualization of functional magnetic resonance neuroimages. Comput Biomed Res.

[43] Cox RW, Chen G, Glen DR, Reynolds RC, Taylor PA (2017). FMRI clustering in AFNI: false-positive rates redux. Brain Connect.

[44] Cole MW, Ito T, Schultz D, Mill R, Chen R, Cocuzza C (2019). Task activations produce spurious but systematic inflation of task functional connectivity estimates. Neuroimage.

[45] Fair DA, Schlaggar BL, Cohen AL, Miezin FM, Dosenbach NUF, Wenger KK (2007). A method for using blocked and event-related fMRI data to study ‘resting state’ functional connectivity. Neuroimage.

[46] Al-Aidroos N, Said CP, Turk-Browne NB (2012). Top-down attention switches coupling between low-level and high-level areas of human visual cortex. Proc Natl Acad Sci USA.

[47] Maier ME, Yeung N, Steinhauser M (2011). Error-related brain activity and adjustments of selective attention following errors. Neuroimage.

[48] McDermott TJ, Wiesman AI, Proskovec AL, Heinrichs-Graham E, Wilson TW (2017). Spatiotemporal oscillatory dynamics of visual selective attention during a flanker task. Neuroimage.

[49] Warm JS, Parasuraman R, Matthews G (2008). Vigilance requires hard mental work and is stressful. Hum Factors.

[50] Pontifex MB, Scudder MR, Drollette ES, Hillman CH (2012). Fit and vigilant: the relationship between poorer aerobic fitness and failures in sustained attention during preadolescence. Neuropsychology.

[51] Iannaccone R, Hauser TU, Staempfli P, Walitza S, Brandeis D, Brem S (2015). Conflict monitoring and error processing: New insights from simultaneous EEG–fMRI. NeuroImage.

[52] Orr C, Hester R (2012). Error-related anterior cingulate cortex activity and the prediction of conscious error awareness. Front Hum Neurosci.

[53] Craig AD (2009). (bud), (Bud) Craig AD. How do you feel — now? The anterior insula and human awareness. Nat Rev Neurosci.

[54] Naqvi NH, Bechara A (2010). The insula and drug addiction: an interoceptive view of pleasure, urges, and decision-making. Brain Struct Funct.

[55] Ikkai A, Curtis CE (2008). Cortical activity time locked to the shift and maintenance of spatial attention. Cereb Cortex.

[56] Kim C, Johnson NF, Gold BT (2012). Common and distinct neural mechanisms of attentional switching and response conflict. Brain Res.

[57] Han SW, Marois R (2014). Functional fractionation of the stimulus-driven attention network. J Neurosci.

[58] Nelson SM, Dosenbach NUF, Cohen AL, Wheeler ME, Schlaggar BL, Petersen SE (2010). Role of the anterior insula in task-level control and focal attention. Brain Struct Funct.

[59] Phillips RC, Salo T, Carter CS (2015). Distinct neural correlates for attention lapses in patients with schizophrenia and healthy participants. Front Hum Neurosci.

[60] Shulman GL, Pope DLW, Astafiev SV, McAvoy MP, Snyder AZ, Corbetta M (2010). Right hemisphere dominance during spatial selective attention and target detection occurs outside the dorsal frontoparietal network. J Neurosci.

[61] Luks TL, Simpson GV, Dale CL, Hough MG (2007). Preparatory allocation of attention and adjustments in conflict processing. Neuroimage.

[62] Franklin T, Wang Z, Suh JJ, Hazan R, Cruz J, Li Y (2011). Effects of varenicline on smoking cue–triggered neural and craving responses. Arch Gen Psychiatry.

[63] Janes AC, Krantz NL, Nickerson LD, Frederick BB, Lukas SE (2020). Craving and cue reactivity in nicotine-dependent tobacco smokers is associated with different insula networks. Biol Psychiatry Cogn Neurosci Neuroimaging.

[64] McClernon FJ, Kozink RV, Rose JE (2008). Individual differences in nicotine dependence, withdrawal symptoms, and sex predict transient fMRI-BOLD responses to smoking cues. Neuropsychopharmacology.

[65] Addicott MA, Sweitzer MM, Froeliger B, Rose JE, McClernon FJ (2015). Increased functional connectivity in an insula-based network is associated with improved smoking cessation outcomes. Neuropsychopharmacology.

[66] Sutherland MT, Carroll AJ, Salmeron BJ, Ross TJ, Hong LE, Stein EA (2013). Individual differences in amygdala reactivity following nicotinic receptor stimulation in abstinent smokers. Neuroimage.

[67] Ding X, Salmeron BJ, Wang J, Yang Y, Stein EA, Ross TJ (2019). Evidence of subgroups in smokers as revealed in clinical measures and evaluated by neuroimaging data: a preliminary study. Addiction Biol.

[68] Hong LE, Hodgkinson CA, Yang Y, Sampath H, Ross TJ, Buchholz B (2010). A genetically modulated, intrinsic cingulate circuit supports human nicotine addiction. Proc Natl Acad Sci USA.

[69] Li S, Yang Y, Hoffmann E, Tyndale RF, Stein EA (2017). CYP2A6 genetic variation alters striatal-cingulate circuits, network hubs, and executive processing in smokers. Biol Psychiatry.

[70] Ashare RL, Falcone M, Lerman C (2014). Cognitive function during nicotine withdrawal: Implications for nicotine dependence treatment. Neuropharmacology.

[71] McClernon FJ, Addicott MA, Sweitzer MM (2015). Smoking abstinence and neurocognition: implications for cessation and relapse. Curr Top Behav Neurosci.

[72] Patterson F, Jepson C, Strasser AA, Loughead J, Perkins KA, Gur RC (2009). Varenicline improves mood and cognition during smoking abstinence. Biol Psychiatry.

[73] Kozink RV, Lutz AM, Rose JE, Froeliger B, McClernon FJ (2010). Smoking withdrawal shifts the spatiotemporal dynamics of neurocognition. Addict Biol.

[74] Dawkins L, Powell JH, West R, Powell J, Pickering A (2007). A double-blind placebo-controlled experimental study of nicotine: II-Effects on response inhibition and executive functioning. Psychopharmacology.

[75] Lesage E, Sutherland MT, Ross TJ, Salmeron BJ, Stein EA (2020). Nicotine dependence (trait) and acute nicotinic stimulation (state) modulate attention but not inhibitory control: converging fMRI evidence from Go–Nogo and Flanker tasks. Neuropsychopharmacology.

